# Opioid response in paediatric cancer patients and the Val158Met polymorphism of the human catechol-O-methyltransferase (COMT) gene: an Italian study on 87 cancer children and a systematic review

**DOI:** 10.1186/s12885-019-5310-4

**Published:** 2019-01-31

**Authors:** Ersilia Lucenteforte, Alfredo Vannacci, Giada Crescioli, Niccolò Lombardi, Laura Vagnoli, Laura Giunti, Valentina Cetica, Maria Luisa Coniglio, Alessandra Pugi, Roberto Bonaiuti, Maurizio Aricò, Sabrina Giglio, Andrea Messeri, Roberto Barale, Lisa Giovannelli, Alessandro Mugelli, Valentina Maggini

**Affiliations:** 1 Department of Clinical and Experimental Medicine University of Pisa, Pisa, Italy; 2 Department of Neuroscience, Psychology, Drug Research and Children’s Health, University of Florence, Florence, Italy; 3 Pain and Palliative Care Unit, Meyer Children’s University Hospital, Florence, Italy; 4 Medical Genetics Unit, Meyer Children’s University Hospital, Florence, Italy; 5 Pediatric Neurology, Neurogenetics and Neurobiology Unit and Laboratories, Neuroscience Department, Meyer Children’s University Hospital, University of Florence, Florence, Italy; 6 Department of Paediatric Oncohematology, Meyer Children’s University Hospital, Florence, Italy; 7 Clinical Trial Office, Meyer Children’s University Hospital, Florence, Italy; 8 Direzione Generale, Azienda Sanitaria Provinciale, Ragusa, Italy; 9 Medical Genetics Unit, Department of Clinical and Experimental Biomedical Sciences “Mario Serio”, University of Florence, Florence, Italy; 10 Department of Biology, University of Pisa, Pisa, Italy; 11 Center for Integrative Medicine, Department of Experimental and Clinical Medicine, Careggi University Hospital, University of Florence, Largo Brambilla, 3 -, 50134 Florence, Italy

**Keywords:** Cancer pain, Children, Opioid, Genetic polymorphisms, Systematic review

## Abstract

**Background:**

Genetic polymorphisms in genes involved in pain modulation have been reported to be associated to opioid efficacy and safety in different clinical settings.

**Methods:**

The association between COMT Val158Met polymorphism (rs4680) and the inter-individual differences in the response to opioid analgesic therapy was investigated in a cohort of 87 Italian paediatric patients receiving opioids for cancer pain (STOP Pain study). Furthermore, a systematic review of the association between opioid response in cancer patients and the COMT polymorphism was performed in accordance with the Cochrane Handbook and the Prisma Statement.

**Results:**

In the 87 paediatric patients, pain intensity (total time needed to reach the lowest possible level) was significantly higher for G/G than A/G and A/A carriers (*p*-value = 0.042). In the 60 patients treated only with morphine, the mean of total dose to reach the same pain intensity was significantly higher for G/G than A/G and A/A carriers (p-value = 0.010). Systematic review identified five studies on adults, reporting that opioid dose (mg after 24 h of treatment from the first pain measurement) was higher for G/G compared to A/G and A/A carriers.

**Conclusions:**

Present research suggests that the A allele in COMT polymorphism could be a marker of opioid sensitivity in paediatric cancer patients (STOP Pain), as well as in adults (Systematic Review), indicating that the polymorphism impact could be not age-dependent in the cancer pain context.

**Trial registration:**

Registration number: CRD42017057831.

**Electronic supplementary material:**

The online version of this article (10.1186/s12885-019-5310-4) contains supplementary material, which is available to authorized users.

## Background

Opioid analgesics are the treatment of choice for moderate-severe pain both in adults and in children [1, 2]. A significant variability both in efficacy and in safety has been observed in many studies aimed at describing inter-individual differences. In addition to demographic and disease-related factors, much research has focused on genetic variability [3]. Indeed, sequence variations in genes involved in opioid pharmacokinetics and pharmacodynamics, as well as in the modulation of pain pathways, have been reported to be associated with parameters of efficacy, such as the dose or the time to obtain analgesia, and of safety, such as the occurrence and severity of opioid side effects, in different clinical settings [3, 4]. Among genes associated with variability in the response to opioid analgesics, the gene encoding for catechol-o-methyltransferase (COMT) is known to be involved in pain modulation, presumably through dopamine-mediated change of enkephalins neuronal content [5], followed in turn by a compensatory regulation of μ-opioid receptors in various brain regions [5, 6].

In particular, the common (50% frequency in Caucasian population) 472G > A single nucleotide polymorphism (SNP) in exon 4 of the COMT gene, Val158Met (rs4680), leading to a three- to four-fold reduced activity of the enzyme [7, 8], has been shown to be associated to higher sensory and regional density of μ-opioid receptors and affect experimental pain ratings [9].

Malignancy- or chemotherapy-induced pain therapy is a challenge for paediatric oncologists. Available information on the impact of genetic variability in COMT on the effect of opioids in cancer pain remains limited, especially in children [10]. In Europe, the incidence rate of cancer in young subjects (0–14 years of age) is 13.9/100,000 [1, 11], with leukemia and nervous system cancer being the most represented (incidence 4.7 and 2.4, respectively). Between 3 and 5% of childhood cancers are bone tumors with osteosarcoma representing the most commonly diagnosed primary malignant cancer, with an incidence rate of 4.0 and 3.1 in young male and female subjects < 24 years, respectively [12].

While pharmacogenetic data on opioids in adult cancer patients are limited, those in paediatric subjects are completely missing. Thus, the aim of Suitable Treatment for Oncologic Pediatric Pain (STOP Pain) study was to investigate the association between the most studied COMT polymorphism, rs4680, and the inter-individual differences in response to opioid analgesic therapy in a cohort of paediatric cancer patients receiving opioids. The opioid dose employed and the modifications in pain intensity were evaluated as efficacy outcomes, and the number of central and gastrointestinal adverse effects as safety outcomes.

Furthermore, to evaluate whether the impact of this COMT polymorphism is age-dependent, data obtained in paediatric patients were compared to existing data in adult subjects. To this aim, a systematic review of published studies was conducted separately for three different outcomes: opioid dosing, pain intensity, and side effects.

## Methods

### STOP Pain study

Materials and methods of the present study have been already published in part by Lucenteforte et al. 2018 [13]. Briefly, STOP Pain is a prospective observational cohort pilot study enrolling hospitalized patients (0–17 years) between June 2011 and December 2014. Being a pilot study, we did not calculate the cohort sample size to test our hypothesis and all recruitable patients were enrolled. The study was approved by the institutional review board of Meyer Children’s Hospital. A psychologist, expert in paediatric pain management, administered to children parents structured questionnaires including demographic information, medical history, concomitant illnesses, and children lifestyle. Data about treatment responsiveness were collected to take in account any confounding variable and/or effect modifier. Patients were anonymized by unique Patient Code, and then matched with genotyping results obtained using the Taqman assay (ABI, Applied Biosystems, Foster City, CA).

Standard opioid conversion to intravenous (IV) morphine equivalents (ME) was performed [13, 14]. The dose of administered morphine was also considered separately by the other drugs. Regarding non-opioid analgesics, the conversion of these medications to IV ME was not performed.

Three indicators of dose were used for pain relief evaluation: cumulative dose (mg/kg) of IV ME administered during the first 24 h of treatment or titration phase (Dose_24h_); total dose (mg/kg) of IV ME from Day 1 to the last day of pain therapy (Dose_tot_); mean dose (mg/kg) required to achieve maximal total pain relief reported by the patient (Dose_VAS = 0_). Pain relief evaluation was also assessed using pain intensity scores. Children pain was measured using three different scales: visual analog scale (VAS) was compiled by 58 subjects over six years of age; Wong & Baker FACES Pain Rating Scale was administered to 5 children between the ages of four and six years of age; Face, Legs, Activity, Cry and Consolability (FLACC) scale was used by nurses to assess pain in 24 children (less than four years old) unable to communicate their pain. These measures were performed at time 0 (before treatment) and defined as PIto. Then, two parameters for pain intensity were considered: difference between the pain intensity after 24 h-treatment and PIto (∆ VAS); time to reach the lowest pain intensity reported by the patient (Time tot). The number of side effects was analyzed using three categorical (presence/absence) variables: onset of any adverse drug reactions (ADRs) and onset of gastrointestinal or CNS effects. Each patient could experience more than one ADR. ADRs were recorded every eight hours for each patient.

### Statistical analyses

Continuous variables were checked for normality by using the Shapiro-Wilk W test. Differences of means for normally distributed variables were compared by one-way ANOVA; differences of medians for non-normally distributed variables were compared by Pearson chi-squared test. Differences of percentages of categorical variables were compared by chi-square test.

We also evaluated association between efficacy (high doses and high pain intensity, defined as values ≥medians, versus low doses and low pain intensity, defined as values <medians) and safety parameters (presence of side effects versus no side effects) and COMT rs4680 polymorphism by calculating odds ratios (ORs) and 95% confidence intervals (CIs) using logistic regression models adjusted for gender, age, body mass index (BMI), diagnosis, metastasis, pain location, and pain intensity at baseline. We considered a *p-value* < 0.05 as significant.

The software STATA 14.2 (StataCorp, 2011; Stata Statistical Software: Release 14. College Station, TX) was used for all analyses.

### Systematic literature review

Cochrane Handbook and the Prisma Statement for Systematic Reviews [15] were used to perform the present review registered in PROSPERO with the number CRD42017057831.

Studies were systematically searched in PUBMED and EMBASE databases up to November 24, 2016 [13]. Full search strategy was reported in Additional file 1. Furthermore, we conducted an additional research for COMT gene to find all possible articles using as search key: COMT[tiab] AND cancer[tiab] AND opioid*[tiab].

Independent investigators (AP and GC) selected articles reviewing titles and abstracts. Two other independent reviewers (EL and VM) resolved through discussion and consensus any disagreements.

Then, AP and GC independently read the full texts selecting the original articles when patients were cancer patients, involved drugs were opioids, outcomes were related to opioid non-response and safety, and one or more genes were studied.

Mean age and gender of the sample, study type and size, location, year of publication, drugs used and genetic information (name of gene, investigated polymorphism and main results) were collected for each study. By study design, only results reported in papers investigating COMT gene as factor associated to therapy outcome were taken into consideration. “Quality Assessment Tool for Observational Cohort and Cross-Sectional Studies” [16] was used to assess the quality of the included studies (see the criteria reported in Additional file 2).

## Results

### STOP Pain study

#### Patient population

One hundred thirty-three patients were evaluated from June 2011 to December 2014. Forty-six children were not included in the study for the following reasons: refusals of consent (*n* = 8), end of life patients (*n* = 12), early discharge (before to administer the questionnaires; *n* = 18), hospitalization in sterile room (n = 8). Table 1 showed the distribution of the main characteristics of the 87 Italian subjects included in the STOP Pain study. Of them, 56% were males. Most were older than 3 years of age and 43% older than 12. BMI was < 25th percentile in 42%, and between 25th and 75th in 26%. Main cancer diagnoses were leukemia or lymphoma (39%), followed by sarcoma (21%) and osteosarcoma (20%). Cancer metastases were present in 26% of the cases; pain in oral cavity in 49%, and skeletal pain in 16%. To achieve pain relief, patients were primarily treated with morphine (69%). A total of five patients (6%) were treated at least once with codeine-containing medication, in particular with a fixed dose combination of codeine and paracetamol. Of them, four patients were administered an oral preparation (codeine 30 mg + paracetamol 500 mg), and only one patient a rectal preparation (codeine 5 mg + paracetamol 200 mg).

**Table 1:** Characteristics of the 87 Italian subjects included in the STOP Pain Project: overall and stratified for COMT rs4680 polymorphism

	Overall	G/G^a^	A/G^a^	A/A^a^	*p-value* ^b^
	N (%)	N (%)	N (%)	N (%)	
	87	24 (30.38)	42 (53.16)	13 (16.46)	
Gender					
Male	49 (56.32)	15 (62.50)	22 (52.38)	7 (53.85)	0.721
Female	38 (43.68)	9 (37.50)	20 (47.62)	6 (46.15)	
Age (months)					
0–36	18 (20.69)	4 (16.67)	8 (19.05)	4 (30.77)	0.466
> 36–144	32 (36.78)	10 (41.67)	18 (42.86)	2 (15.38)	
> 144	37 (42.53)	10 (41.67)	16 (38.10)	7 (53.85)	
BMI (percentile)					
< 25th	33 (42.31)	5 (25.00)	18 (46.15)	5 (38.46)	0.490
25th- < 75th	20 (25.64)	8 (40.00)	8 (20.51)	4 (30.77)	
≥ 75th	25 (32.05)	7 (35.00)	13 (33.33)	4 (30.77)	
*missing*	*9*				
Diagnosis					
Brain Tumour	6 (6.90)	2 (8.33)	2 (4.76)	–	0.728
Leukaemia and Lymphoma	34 (39.08)	11 (45.83)	18 (42.86)	4 (30.77)	
Neuroblastoma	6 (6.90)	1 (4.17)	3 (7.14)	2 (15.38)	
Osteosarcoma	17 (19.54)	2 (8.33)	8 (19.05)	3 (23.08)	
Sarcoma	18 (20.69)	7 (29.17)	8 (19.05)	2 (15.38)	
Others	6 (6.90)	1 (4.17)	3 (7.14)	2 (15.38)	
Metastasis					
No	64 (73.56)	19 (79.17)	26 (61.90)	12 (92.31)	0.067
Yes	23 (26.44)	5 (20.83)	16 (38.10)	1 (7.69)	
Pain location					
Abdominal	12 (13.79)	5 (20.83)	4 (9.52)	2 (15.38)	0.808
Oral cavity	43 (49.43)	11 (45.83)	23 (54.76)	6 (46.15)	
Skeletal – Muscle	14 (16.09)	3 (12.50)	5 (11.90)	3 (23.08)	
Other	18 (20.69)	5 (20.83)	10 (23.81)	2 (15.38)	
Pain Intensity (PI_to_), mean (95% CI)	4.34 (3.88–4.81)	4.08 (3.33–4.84)	4.57 (3.89–5.25)	4.38 (2.81–5.96)	0.674^a^
Drug					
morphine	60 (68.97)	16 (66.67)	32 (76.19)	8 (61.54)	
tramadol	19 (21.84)	5 (20.83)	9 (21.43)	3 (23.08)	0.632
oxycodone	2 (2.30)	1 (4.17)	–	–	
codeine	2 (2.30)	1 (4.17)	–	1 (7.69)	
more than one	4 (4.60)	1 (4.17)	1 (2.38)	1 (7.69)	

^a^ Biological samples of 8 patients were not available for medical reasons. Genetic data were not available for technical reasons (failure of the genetic test)

^b^ *p*-value from ANOVA

Biological samples of eight patients were not available for medical reasons, however main characteristics of these patients were comparable with those of genotyped patients (see Additional files 3 and 4).

#### Influence of COMT polymorphism on efficacy and safety parameters

None of the above characteristics was different among the three genotype groups (*p-values* ≥ 0.05). Genotype frequencies agreed with the Hardy–Weinberg equilibrium (*p-value* = 0.45) and consistent with 1000 Genome Project Data for European population (EUR) [17].

Table 2 shows mean and standard deviation values of opiate and morphine dose, pain intensity, and distribution of side effects in the three genotype groups. The mean of Time_tot_ significantly differed across the three groups (*p-value* = 0.042). In particular, the mean for AG and AA groups was lower than GG (*p-value* = 0.0094 and 0.0026, respectively). No difference was observed for all the other considered variables (*p-values* ≥ 0.05).

**Table 2:** Efficacy and safety parameters according to COMT rs4680 polymorphism in the 87 subjects included in the STOP Pain Project

	Overall	G/G	A/G	A/A	*p-value*
Opioids					
Dose (mg/kg)					
Dose_24h_					
mean (95% CI)	0.38 (0.33–0.42)	0.41 (0.30–0.52)	0.39 (0.33–0.45)	0.36 (0.23–0.48)	
median (interquartile range)	0.41 (0.19–0.50)	0.43 (0.16–0.57)	0.42 (0.24–0.49)	0.43 (0.19–0.46)	0.921^*^
Dose_tot_					
mean (95% CI)	2.57 (2.17–2.96)	3.34 (2.45–4.23)	2.35 (1.83–2.87)	2.24 (1.11–3.37)	
median (interquartile range)	2.18 (1.20–3.50)	3.25 (1.73–5.23)	2.02 (1.26–2.97)	2.18 (1.06–3.07)	0.119^*^
Dose_VAS = 0_					
mean (95% CI)	0.41 (0.27–0.55)	0.59 (0.18–1.01)	0.35 (0.22–0.48)	0.39 (0.03–0.75)	
median (interquartile range)	0.26 (0.10–0.49)	0.36 (0.07–0.74)	0.21 (0.11–0.47)	0.25 (0.10–0.41)	0.568^*^
Pain Intensity					
∆ _VAS,_ N (%)					
≤ 2	46 (54.12)	15 (62.50)	20 (50.00)	6 (46.15)	0.533^**^
≥ 2	39 (45.88)	9 (37.50)	20 (50.00)	7 (53.85)	
Time _tot_ (hours)					
mean (95% CI)	140.43 (126.81–154.05)	166.87 (143.42–190.33)	133.67 (111.87–155.46)	116.61 (87.64–145.59)	0.042^***^
median (interquartile range)	133.00 (99.00–192.00)	190.50 (139.25–199.00)	130.00 (96.00–185.50)	120.00 (76.00–144.00)	
Side effects, N (%)^a^					
Gastrointestinal^b^	23 (26.44)	8 (33.33)	9 (21.43)	3 (23.08)	0.553^**^
CNS^c^	10 (11.49)	2 (8.33)	4 (9.52)	2 (15.38)	0.780^**^
Total^d^	32 (36.78)	10 (41.67)	14 (33.33)	4 (30.77)	0.736^**^
Morphine					
Dose (mg/kg)					
Dose_24h_					
mean (95% CI)	0.49 (0.45–0.53)	0.55 (0.44–0.66)	0.47 (0.41–0.52)	0.49 (0.38–0.60)	
median (interquartile range)	0.46 (0.41–0.57)	0.50 (0.43–0.65)	0.46 (0.40–0.55)	0.46 (0.43–0.60)	0.170^*^
Dose_tot_					
mean (95% CI)	3.19 (2.72–3.67)	4.39 (3.49–5.30)	2.75 (2.14–3.35)	3.04 (1.48–4.60)	
median (interquartile range)	2.93 (1.82–4.42)	4.52 (3.25)	2.23 (1.72–3.40)	2.98 (1.70–3.33)	0.050^*^
Dose_VAS = 0_					
mean (95% CI)	0.54 (0.35–0.74)	0.88 (0.28–1.49)	0.41 (0.25–0.56)	0.56 (0.00–1.16)	
median (interquartile range)	0.36 (0.17–0.60)	0.54 (0.36–0.96)	0.26 (0.14–0.52)	0.37 (0.20–0.48)	0.257^*^
Pain Intensity					
∆ _VAS,_ N (%)					
≤ 2	29 (49.15)	10 (62.50)	16 (51.61)	2 (25.00)	0.221^**^
≥ 2	30 (50.85)	6 (37.50)	15 (48.39)	6 (75.00)	
Time _tot_ (hours)					
mean (95% CI)	147.96 (131.56–164.36)	174.62 (149.82–199.43)	139.53 (113.56–165.50)	134.00 (96.72–171.28)	0.154^***^
median (interquartile range)	142.50 (101.50–193.50)	190.50 (150.00–199.00)	130.25 (100.5–188.75)	123.50 (99.50–173.25)	
Side effects, N (%)^a^					
Gastrointestinal^b^	14 (23.33)	5 (31.25)	6 (18.75)	2 (25.00)	0.621^**^
CNS^c^	6 (10.00)	1 (6.25)	2 (6.25)	1 (12.50)	0.817^**^
Total^d^	19 (31.67)	6 (37.57)	9 (28.13)	2 (25.00)	0.752^**^

Opioids: morphine equivalents (patients that used more than one opioid)

Morphine: patients that used only morphine

Dose_24h_: total dose (mg/kg) of intravenous (IV) morphine equivalents (ME) administered during the titration phase; Dose_tot_: total dose (mg/kg) of IV ME; Dose_VAS = 0_: mean dose (mg/kg) required to achieve total pain relief

PI_to_: pain intensity before treatment, measured with FLACC (Face, Legs, Activity, Cry, Consolability) or VAS (Visual Analogic Scale) numeric scale or WONG & BAKER Pain Rating Scale (range: 0–10); ∆ _VAS_: difference between the pain intensity after 24 h of treatment and PI_to_; Time _tot_: time in hours to reach the lowest pain intensity possible

^a^ N, number of patients who experienced that ADR; each patient could have experienced more than one ADRs

^b^ Gastrointestinal effects included nausea/vomiting, diarrhea, and constipation

^c^ CNS: Central Nervous System effects included agitation, drowsiness, headache, and sedation

^d^ Total number of patients with gastrointestinal and/or central nervous system effects, and/or itching

^*^ *p*-value from Pearson chi-squared test of the equality of the medians

^**^ *p*-value form ANOVA

^***^ *p*-value from chi-squared test

Table 3 shows the association of efficacy and safety parameters with COMT rs4680 polymorphism. After the adjustment for gender, age, BMI, diagnosis, metastasis, pain location and pain intensity at baseline, we found a statistically significant association for the efficacy parameters Dose_tot_ and Time_tot_. In particular, A/G subjects needed a lower total amount of drugs compared to G/G individuals (adjusted OR 0.27, 95% CI 0.08–0.87), and patients heterozygous or homozygous for the A allele reached the lowest pain intensity faster compared to G/G individuals (OR 0.18, 95% CI 0.05–0.63 and OR 0.11, 95% CI 0.02–0.56, respectively). No significance was found for the association of safety parameters and COMT genotype.

**Table 3:** Association of efficacy and safety parameters with COMT rs4680 polymorphism in the 87 subjects included in the STOP Pain Project

	N (%) OR^a^ (95% CI)		
	G/G	A/G	A/A
Dose (mg/kg)			
High Dose_24h_ (≥0.41 vs < 0.41)	12 (50.00) vs 12 (50.00)	21 (52.50) vs 19 (47.50)	7 (53.85) vs 6 (46.15)
	1 (reference)	0.66 (0.19–2.25)	0.80 (0.16–4.01)
High Dose_tot_ (≥2.18 vs < 2.18)	16 (66.67) vs 8 (33.33)	18 (42.86) vs 24 (57.14)	7 (53.85) vs 6 (46.15)
	1 (reference)	0.27 (0.08–0.87)	0.42 (0.09–1.98)
High Dose_VAS = 0_ (≥0.26 vs < 0.26)	14 (58.33) vs 10 (41.67)	26 (65.00) vs 14 (35.00)	9 (69.23) vs 4 (30.77)
	1 (reference)	0.98 (0.30–3.24)	1.47 (0.29–7.40)
Pain Intensity			
High ∆_VAS_ (≥2 vs < 2)	14 (58.33) vs 10 (41.67)	20 (47.62) vs 22 (52.38)	6 (46.15) vs 7 (53.85)
	1 (reference)	0.51 (0.15–1.65)	0.57 (0.12–2.82)
High Time_tot_ (≥133 vs < 133)	19 (79.17) vs 5 (20.83)	19 (45.24) vs 23 (54.76)	4 (30.77) vs 9 (69.23)
	1 (reference)	0.18 (0.05–0.63)	0.11 (0.02–0.56)
Side effects, N (%)			
Gastrointestinal^b^	8 (33.33) vs 16 (66.67)	9 (21.43) vs 33 (78.57)	3 (23.08) vs 10 (76.92)
	1 (reference)	0.56 (0.16–1.91)	0.46 (0.09–2.44)
CNS^c^	2 (8.33) vs 22 (91.67)	4 (9.52) vs 38 (90.48)	2 (15.38) vs 11 (84.62)
	1 (reference)	1.39 (0.21–9.44)	1.62 (0.17–15.78)
Total^d^	10 (58.33) vs 10 (41.67)	14 (33.33) vs 28 (66.67)	4 (30.77) vs 9 (69.23)
	1 (reference)	0.86 (0.29–2.56)	0.58 (0.13–2.54)

Dose_24h_: total dose (mg/kg) of intravenous (IV) morphine equivalents (ME) administered during the titration phase; Dose_tot_: total dose (mg/kg) of IV ME; Dose_VAS = 0_: mean dose (mg/kg) required to achieve total pain relief

PI_to_: pain intensity before treatment, measured with FLACC (Face, Legs, Activity, Cry, Consolability) or VAS (Visual Analogic Scale) numeric scale or WONG & BAKER Pain Rating Scale (range: 0–10); ∆ _VAS_: difference between the pain intensity after 24 h of treatment and PI_to_; Time _tot_: time in hours to reach the lowest pain intensity possible

^a^OR and corresponding 95% confidence intervals from logistic regression models adjusted for gender, age, BMI, diagnosis, metastasis, pain location and pain intensity at baseline

^b^Gastrointestinal side effects comprehend nausea, vomiting, diarrhea and constipation

^c^CNS, Central Nervous System side effects comprehend agitation, drowsiness, headache and sedation

^d^Total side effects comprehend the occurrence of gastrointestinal and CNS side effects, and itching

### Systematic literature review

#### Study selection

**Fig. 1:**
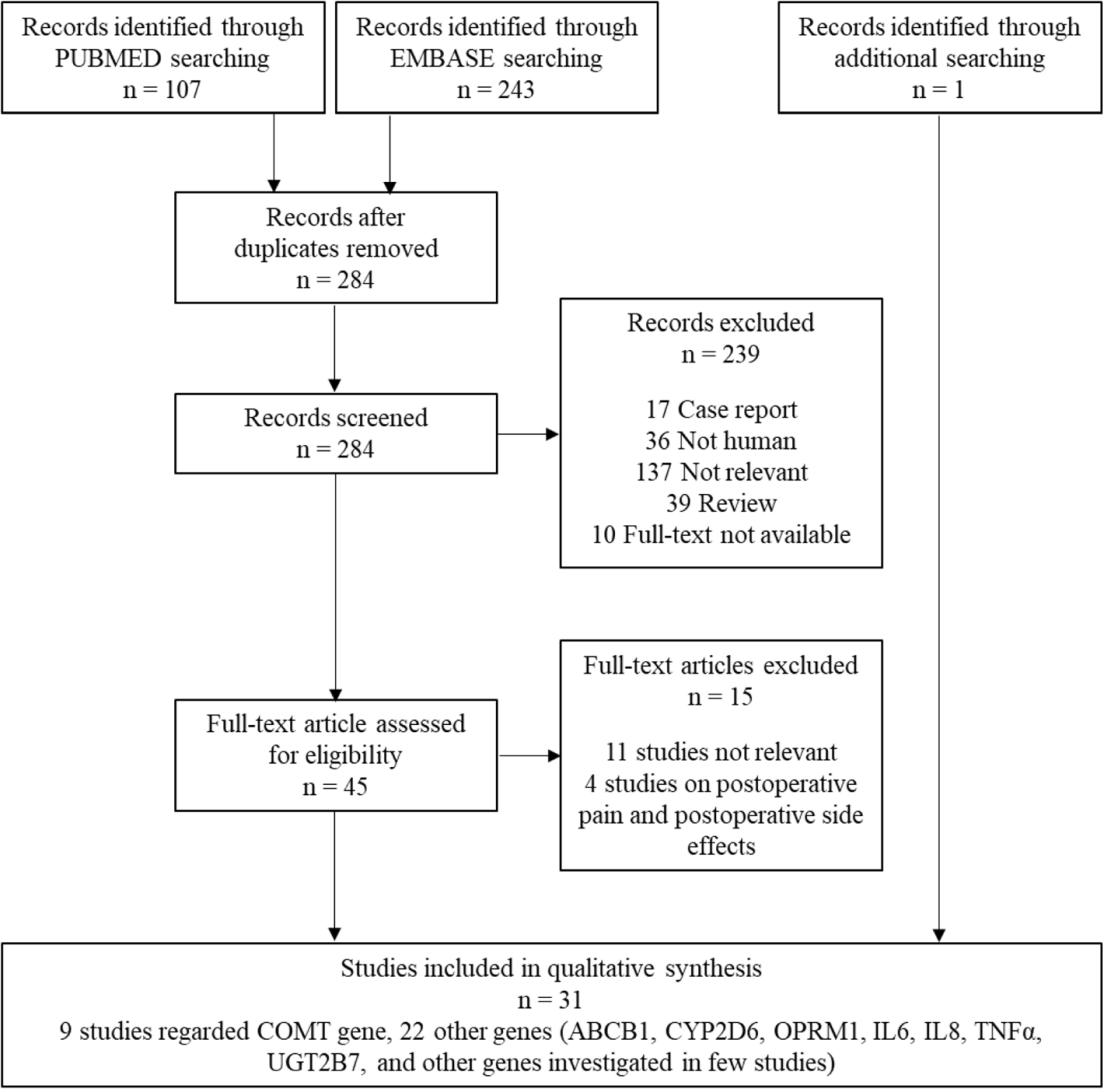
PRISMA Flow diagram of the selection of studies searched with PUMBED and EMBASE and included in the systematic review

Systematic literature search produced 350 records. After an initial screening of titles and abstracts, 239 studies were excluded for the following reasons: not relevant (137); reviews (39); not human studies (36 studies); case reports (17); full text not available (10). We obtained the full text of 45 articles. Of them, 11 studies were defined not relevant because they did not focus on genotype effects on our clinical outcomes (pain relief and adverse events), and four focused on cancer patients’ postoperative pain and postoperative adverse events, thus they were excluded. Characteristics of studies excluded after full-text reading are shown in Additional file 5. Finally, we included a total of 31 studies (see the flowchart of study selection in Fig. 1): 30 from the above 45 studies and 1 additional study obtained with further search strategy (identifying 16 records).

#### Study quality assessment

Of the 31 selected papers, nine investigated COMT and were analyzed in the present review [18–26]; none of the studies was conducted on paediatric subjects. Assessment of methodological quality was performed for the considered studies using the Quality Assessment Tool for Observational Cohort and Cross-Sectional Studies [16]. All studies met the quality criteria regarding the research question or objective (items 1), inclusion/exclusion criteria and participants’ recruitment (item 4), exposure of interest measurement prior to the outcome measurement (item 6), length of follow-up (item 7), independent and dependent variable description/definition (item 9 and 11), multiple assessment of the exposure (item 10), and lost at follow-up (item 13). Only 28 and 33% of studies received negative responses for the items 2 and 14, respectively. Participation rate of eligible patients (items 3) and sample size justification (item 5) were clearly described in 42 and 8% of studies, respectively. Categories of exposure (item 8) and blinding of assessors (item 12) were applicable only in 22 and 3% of studies, respectively.

#### Study description

Table 4 shows the characteristics of five studies (reported in nine papers) included in the systematic review. Three studies were conducted in Europe, one in Japan, and one in Tunisia. The EPOS study included the highest number of patients; drugs used were morphine in three studies, morphine, methadone, fentanyl, hydromorphone, buprenorphine, ketobemidone, and oxycodone in another and morphine, oxycodone, fentanyl, and methadone in the last study. Three studies reported data on opioid dose as well as on pain and side effects.

**Table 4:** Characteristics of five studies (nine papers) investigating the association between COMT gene and opioid response and/or side effects included in the systematic review

Study name, [ref]	Study design	Patients characteristics	Opioid administered	Data			M/F	Mean Age (years)
				Opioid dose	Pain	Side effects		
EPOS study	European observational study	Cancer pain patients	Morphine, methadone, fentanyl, hydromprphone, buprenorphine, ketobemidone, oxycodone					
[18] (Klepstad, 2011)		2201 Caucasians		X			1154/1047	62.4
[19] (Barratt, 2014)^a^		667 subjects treated with transdermal fentanyl					334/342	Median: 64
[20] (Barratt, 2015)^b^		468 Caucasian subjects treated with transdermal fentanyl			X	X	218/250	Median: 64
[21] (Laugsand, 2011)		1579 subjects not receiving chemotherapy and with information on nausea and vomiting				X	850/729	61.9
[22] Matsuoka, 2012	Japanese observational study	48 Opioid-treatment-naïve cancer patients	Morphine	X			25/23	69.0
[23, 24] Rakvåg, 2005–2008	Norwegian observational study	207 Cancer pain Caucasian patients	Morphine	X	X	X	117/90	63.2
[28] Ross, 2008	United Kingdom case-control study	228 Cancer pain patients	Morphine, Oxycodone, fentanyl, methadone		X	X	106/122	57.2
[26] Chatti, 2016	Tunisian observational study	129 Cancer pain patients	Morphine	X			63/66	Number of patients for each age group: 17-25 yrs.: 12 26-45 yrs.: 50 46-65 yrs.: 67

^a^Barratt, 2014 [19] was a subgroup analysis of Klepstad, 2011 [18] (i.e. 676 subjects treated with transdermal fentanyl)

^b^Barratt, 2015 [20] was a subgroup analysis of Barratt, 2014 [19] (i.e. 468 Caucasian subjects treated only with transdermal fentanyl)

Tables 5, 6 and 7 report the evaluated association between opioid dose, pain intensity, and side effects with *COMT* rs4680 polymorphism. Two studies included in the systematic review agreed on the finding that opioid Dose_24h_ was lower among subjects with the A (158 Met) allele [22, 23]. Only one study [26] reported an association between the Dose_tot_ of oral morphine and dose escalation, without reaching statistical significance. Otherwise, no clear associations emerged with pain intensity (measured with Brief Pain Inventory) or with side effects, with the exception of the EPOS study in which the intensity of nausea and vomiting (EORTC score) was significantly lower among the subjects with A/G genotype (*p-value* = 0.002).

**Table 5:** Association between opioid dose and COMT rs4680 polymorphism in the studies included in the systematic review

Study name [ref]	Variable	Genotype frequency (N, %)	Results (type of measure)	*p-value*
EPOS study [18] (Klepstad, 2011)	Dose in mg after 24 h	G/G (324, 22.18) A/G (726, 49.69) A/A (411, 28.13)	180 mg 180 mg 160 mg (median)	0.545
[22] Matsuoka, 2012	Dose in mg after 24 h	G/G (19, 46.34) A/G (18, 43.90) A/A (4, 9.76)	43.7 ± 21.4 28.9 ± 3.2 30.0 ± 0.0 (mean ± SD)	0.03
[23] Rakvåg, 2005	Dose in mg after 24 h	G/G (44, 21.25) A/G (96, 46.38) A/A (67, 32.37)	155 ± 160 117 ± 100 95 ± 99 (mean ± SD)	0.025
[26] Chatti, 2016	Total dose requirement (continuous) AvsG	G/G (30, 23.3) A/G (57, 44.2) A/A (42, 32.6)	−2.10 (difference)	0.334
	Need of escalation (yes/no) AvsG		OR 0.76 (0.45; 1.27)	0.293

**Table 6:** Association between pain intensity and COMT rs4680 polymorphism in the studies included in the systematic review

Study name [ref]	Variable	Genotype frequency (N, %)	Results (type of measure)	*p-value*
EPOS study [20] (Barratt, 2015)	Brief Pain Inventory	G/G (109, 23.59) A/G (243, 52.60) A/A (110, 23.80)	Not reported	
[23] Rakvåg, 2005	Brief Pain Inventory after 24 h	G/G (44, 21.25) A/G (96, 46.38) A/A (67, 32.37)	3.9 ± 2.2 3.7 ± 2.6 3.5 ± 2.3 (mean ± SD)	> 0.05
[28] Ross, 2008	Brief Pain Inventory after 24 h	G/G (46, 20.81) A/G (119, 53.85) A/A (56, 25.34)	Not reported	0.897

**Table 7:** Association between side effects and COMT rs4680 polymorphism in the studies included in the systematic review

Study name [ref]	Variable	Genotype frequency (N, %)	Results (type of measure)	*p-value*
EPOS study [20] (Barratt, 2015)	Tiredness, Depression, Cognitive Dysfunction, Constipation	G/G (109, 23.59) A/G (243, 52.60) A/A (110, 23.80)	Not reported	
[21] (Laugsand, 2011)	Nausea and Vomiting EORTC Score	G/G (341, 21.93) A/G (787, 50.61) A/A (427, 27.46)	25.8 ± 30.5 21.5 ± 26.6 26.2 ± 29.2 (mean ± SD)	0.002
[23] Rakvåg, 2005	Fatigue EORTC Score	G/G (44, 21.25) A/G (96, 46.38) A/A (67, 32.37)	73 ± 23 62 ± 24 66 ± 22 (mean ± SD)	> 0.05
	Nausea and vomiting EORTC Score		30 ± 27 24 ± 26 29 ± 27 (mean ± SD)	> 0.05
	Dyspnea EORTC Score		39 ± 35 38 ± 34 32 ± 33 (mean ± SD)	> 0.05
	Sleep EORTC Score		39 ± 35 38 ± 34 32 ± 33 (mean ± SD)	> 0.05
	Appetite EORTC Score		64 ± 36 49 ± 38 53 ± 38 (mean ± SD)	> 0.05
	Constipation EORTC Score		56 ± 41 57 ± 37 54 ± 37 (mean ± SD)	> 0.05
[28] Ross, 2008	Central side effect	G/G (46, 20.81) A/G (119, 53.85) A/A (56, 25.34)	Not reported	0.956

*EORTC* European Organization for Research and Treatment of Cancer Core

Prisma checklist validation is reported in Additional file 6.

#### Comparison of STOP PAIN and systematic review results

**Fig. 2:**
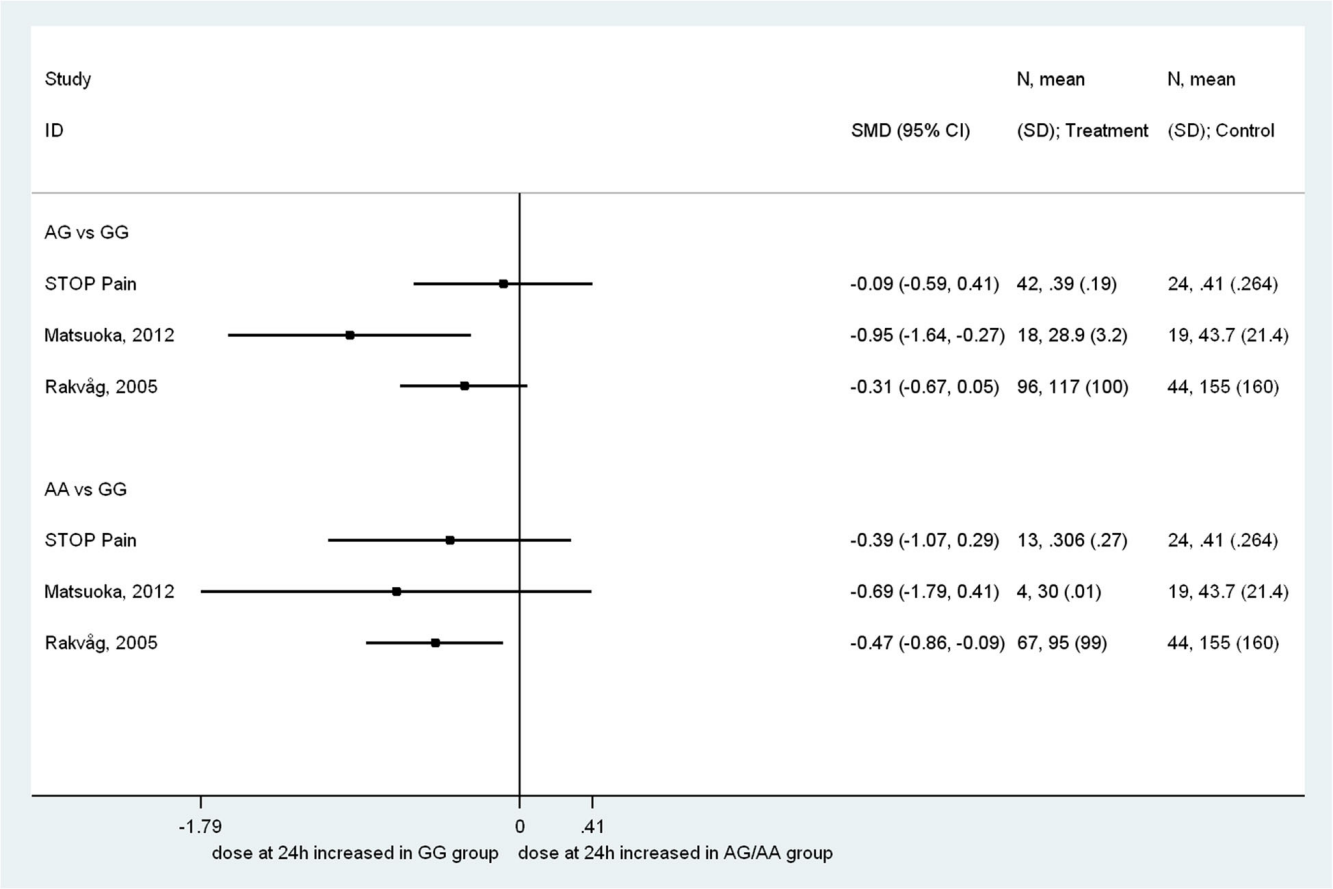
Forest plot of the comparison of STOP Pain data with those included in the systematic review, relative to the 24-h opioid cumulative dose (when reported as mean ± SD)

Figure 2 allows a direct visualization via a forest plot of the comparison of the STOP PAIN data with those included in the systematic review, relative to the 24 h opioid cumulative dose (when reported as mean ± standard deviation (SD)).

## Discussion

This is the first paediatric study addressing the role of COMT polymorphism rs4680 in opioid treatment of cancer patients as well as the inter-individual differences in the response to opioid analgesic therapy. Furthermore, we also compared the obtained evidences to existing data in adults retrieved by means of a systematic review of medical literature. The main result of the STOP Pain study was that paediatric cancer patients with the GG genotype needed a longer time to reach the lowest possible pain intensity compared with the A-containing genotypes. However, this association can be due to chance, given the many interaction tests carried out. The systematic review regarding adult cancer patients showed that the Dose24h was lower among subjects presenting the A allele [22, 23, 27], and no clear association was found between the polymorphism and either pain intensity or side effects.

Focusing on investigations into the role of COMT rs4680 polymorphism in cancer pain, the G/G genotype was shown to require higher morphine daily doses as compared to G/A and A/A ones [22, 23, 27]. This association was not confirmed in Caucasian adults by a large multicenter European (11 countries) study [18] and an investigation on Tunisian patients [26]. Genetic heterogeneity (and cosmopolitan areas) could contribute to this contradictory finding compared to the others homogenous populations [22, 23].

On the other hand, the first study showed an association of this polymorphism with opioid-induced nausea [21]. In another study, morphine central side effects were shown to be associated with a different COMT polymorphism [28]. The relevance of the selected candidate gene was due to the association between the catechol-O-methyltransferase and chronic pain: COMT affects dopamine concentration in the prefrontal cortex of the human brain, influencing pain regulation at different levels [29]. Several of the most frequent SNPs within COMT have been investigated in relation to mRNA expression, protein levels and enzyme activity. Principally, even if a more complex genetic basis could not be excluded, the nonsynonymous SNP rs4680 variation induced decreased enzyme thermostability and activity [29]. This lower activity of COMT enzyme, with a consequent increase of dopamine bioavailability [4], was reported to increase opioid receptor density and to enhance opioid analgesia and adverse effects in several types of cancer pain [18, 21–23].

COMT polymorphisms associated with lower COMT activity have been studied in several painful conditions, such as postoperative surgery, cancer pain, neuropathic pain, and migraine or headache [30], not only in adult subjects but also in paediatric populations [31–34]. In osteoarthritis, the low-activity allele of COMT [158Met or A] was associated with increased hip pain in patients with damaged hip [35]. Low COMT activity has been related to increased pain sensitivity in experimental tests, whereas high activity COMT haplotypes protect from the development of chronic muscle-skeletal painful conditions [36].

In our sample of paediatric patients, the association with rs4680 *COMT* polymorphism was found using the total dose requested for maximal possible pain reduction, whereas the 24-h dose that correlated in adult cancer patients only showed a slight tendency towards association. The reasons for this discrepancy may reside in differences between adults and children in both pharmacokinetics and pharmacotherapy regimens. In fact, while pharmacological therapy in adults is based on fixed-dose increments, treatment in children needs to be personalized based on age and weight (or body surface), considering incomplete organ maturation, differences in body composition and plasma proteins, in receptor density and type. For example, children younger than 11 years show faster clearance of morphine and morphine metabolites than older subjects [37]. Furthermore, there is wide variability in morphine clearance among paediatric subjects, particularly neonates and infants [38].

For these patients, analgesics are the drug class most commonly used in hospital [39], with morphine being one of the most used among the strong opioids [40], and some of these drugs (i.e., oxycodone, tramadol) are generally used in an off-label manner, with a potential increased risk of adverse effects. Moreover, experimental and clinical data support the presence of significant differences between opioid pharmacological characteristics [41]. Although all opioids interact with the mu (μ) opioid receptor as primary target, these compounds differ in chemical structures, in pharmacokinetics, efficacy, effectiveness, and toxicity [42]. Moreover, the use of equianalgesic tables raises several concerns: (1) calculating the median equivalence values based on them may be inaccurate, mainly for possible different variable equivalence ranges within or between different equianalgesic tables; (2) equivalence ratios derived from computation rather than from clinical data could be devoid of clinical context and might be grossly inaccurate; (3) formulations containing an opioid and other analgesics (i.e., nonsteroidal anti-inflammatory drugs) cannot be compared to a single opioid and calculation of equivalents may be difficult [43].

Overall, results obtained for adults and children in both the total amount of opioids administered and in the dose administered during the first 24 h, could be primarily related to the factors mentioned above: developmental pharmacokinetic, medications’ differences, and the use of conversion tables. These variables could have influenced the relationship between COMT polymorphism and the opioids doses.

In light of these considerations, the secondary aim of the present study (whether the impact of rs4680 polymorphism is age-dependent) is not completely pointed out. Our results suggest that, independently from age, rs4680 could actively influence the efficacy of pain therapy also in children. Future studies, with large number of paediatric patients, are needed to fully ascertain the trend towards association shown also by our observational data.

Current guidelines encourage accurate monitoring of pain in children and its treatment [2] in order to reduce as much as possible, the psychological and behavioral effects of the pain sensation, whose impact is greater in subjects during growth [44]. However, pain perception and ability to report it is not homogeneous within the paediatric population making it necessary to use different pain evaluation scales, which for younger subjects rely only on signs: this can be another factor concurring to increase the inter-individual variation in the requested opioid dose, particularly during the initial titration phase. Furthermore, the inclusion of different pain rating scales raises the problem of consistency between the scales, although FLACC scores were reported to be comparable to those generated using 0-to-10 number rating scales [45], and pain severity ratings with the Wong & Baker scale resulted highly correlated with those of VAS [46].

The number of enrolled patients in the STOP Pain study was relatively small. However, the uniform treatment regimen, with 69% of patients receiving titration of morphine by continuous *i.v.* infusion, reduces the effect of this shortcoming. Future studies should prospect a multi-center collaborative setting to address this point. Moreover, regarding pharmacological treatment, we could not establish whether any non-opioid analgesics used in our sample may have affected pain ratings and side effects. This information would be very important, since some agents may synergize with opioids and/or have similar genetic influences on metabolism and/or drug sensitivity.

Another limitation is represented by the mixed nature of pain in cancer patients. In fact, paediatric cancer pain can vary from acute, procedure-related pain to progressive chronic and breakthrough pain, associated to disease progression and/or treatment. Moreover, due to the relatively small sample size when considering the wide variability of cancer diagnoses, it was difficult to state whether the type and location of the primary cancer or metastases influenced the pain measurements independent of genetic influences on opioid metabolism and receptor activity. Thus, whether cancer-associated pain may represent an additional bias, it may only be determined through a large study including comparison of patients with pain of different origins. Finally, an additional obvious limitation is that the present results in children can only be compared with those reported in studies conducted on adults, simply due to the lack of similar studies in paediatric subjects. On the other hand, comparison of paediatric data with those obtained in adults, collected in a systematic review, has shown differences in the relevant parameters (24 h dose vs. total dose) providing useful information for future studies in children.

## Conclusions

The results of this research show that paediatric cancer patients with the GG genotype, compared with the A-containing genotypes, received a higher mean dose of morphine equivalents and needed a longer time to reach the lowest possible pain intensity. These results suggest that the presence of A allele in COMT rs4680 SNP could represent an evaluable marker of opioid sensitivity in paediatric cancer patients, as well as in adults. Although further studies are needed to confirm these findings, to date these evidences are still not sufficient to support a previous expensive evaluation of COMT SNPs before starting an opioid therapy in children suffering for cancer pain.

## Additional files

Additional file 1: BMC Cancer.doc, Full Search Strategy. (DOCX 26 kb)

Additional file 2: BMC Cancer.doc, Criteria for the quality assessment of the included studies in the review. (DOCX 272 kb)

Additional file 3: BMC Cancer.doc, Characteristics of 8 missing subjects in the STOP Pain Project. (DOCX 24 kb)

Additional file 4: BMC Cancer.doc, Efficacy and safety parameters of 8 missing subjects in the STOP Pain Project. (DOCX 25 kb)

Additional file 5: BMC Cancer.doc, Characteristics of studies excluded after full-text reading. (DOCX 26 kb)

Additional file 6: BMC Cancer.doc, PRISMA Checklist for the current review. (DOCX 30 kb)

## Abbreviations

ADRs: Adverse drug reaction
BMI: Body mass index
CIs: Confidence intervals
COMT: Catechol-O-methyltransferase
FLACC: Face, Legs, Activity, Cry and Consolability
IV: Intravenous
ME: Morphine equivalents
ORs: Odds ratios
SD: Standard deviation
SNP: Single nucleotide polymorphism
STOP PAIN: Suitable Treatment for Oncologic Pediatric Pain
VAS: Visual analog scale
