# Supplementary figures and images for: Opioid response in paediatric cancer patients and the Val158Met polymorphism of the human catechol-O-methyltransferase (COMT) gene: an Italian study on 87 cancer children and a systematic review
Source: BMC Cancer. 2019 Jan 31;19:113. doi: 10.1186/s12885-019-5310-4 (PMC6357360; doi:10.1186/s12885-019-5310-4)

**Table S2.** Criteria for the quality assessment of the included studies in the review.


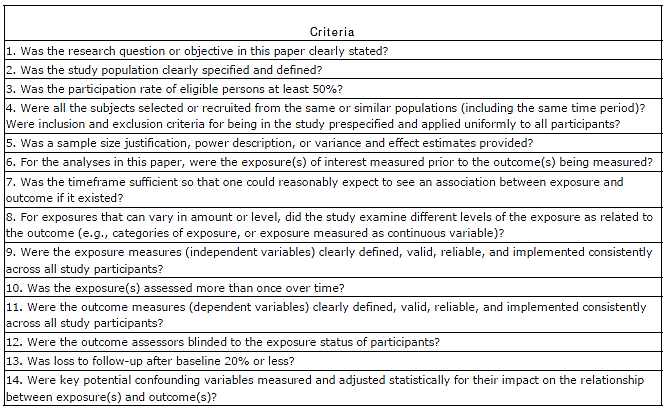

Supplement: Supplementary file 2 — BMC Cancer.doc, Criteria for the quality assessment of the included studies in the review. (DOCX 272 kb) [file 12885_2019_5310_MOESM2_ESM.docx]
