# Supplementary material for: Opioid response in paediatric cancer patients and the Val158Met polymorphism of the human catechol-O-methyltransferase (COMT) gene: an Italian study on 87 cancer children and a systematic review
Source: BMC Cancer. 2019 Jan 31;19:113. doi: 10.1186/s12885-019-5310-4 (PMC6357360; doi:10.1186/s12885-019-5310-4)
Supplement: Supplementary file 3 — BMC Cancer.doc, Characteristics of 8 missing subjects in the STOP Pain Project. (DOCX 24 kb) [file 12885_2019_5310_MOESM3_ESM.docx]

**Table S3.** Characteristics of 8 missing subjects in the STOP Pain Project.

|  | **Overall** |
| --- | --- |
|  | **N (%)** |
|  | 8 |
| **Gender** |  |
| Male | 5 (62.50) |
| Female | 3 (37.50) |
| **Age** (months) |  |
| 0-36 | 2 (25.00) |
| >36-144 | 2 (25.00) |
| >144 | 4 (50.00) |
| **BMI (percentile)** |  |
| <25th | 2 (25.00) |
| 25th-<75th | 2 (25.00) |
| ≥75th | 2 (25.00) |
| *missing* | *2* |
| **Diagnosis** |  |
| Brain Tumour | 2 (25.00) |
| Leukaemia and Lymphoma | -- |
| Neuroblastoma | 1 (12.50) |
| Osteosarcoma | -- |
| Sarcoma | 4 (50.00) |
| Others | 1 (12.50) |
| **Metastasis** |  |
| No | 7 (87.50) |
| Yes | 1 (12.50) |
| **Pain location** |  |
| Abdominal | 1 (12.50) |
| Oral cavity | 3 (37.50) |
| Skeletal - Muscle | 3 (37.50) |
| Other | 1 (12.50) |
| **Pain Intensity** (PI_to_)  Mean (±SD) | 3.87 (±2.571) |
| **Pain Intensity** (PI_to_) **grouped** |  |
| ≤4 | 5 (62.50) |
| >4 | 3 (37.50) |
| **Drug** |  |
| morphine | 4 (50.00) |
| tramadol | 2 (25.00) |
| oxycodone | 1 (12.50) |
| codeine | -- |
| more than one | 1 (12.50) |
|  |  |
