# Supplementary material for: Opioid response in paediatric cancer patients and the Val158Met polymorphism of the human catechol-O-methyltransferase (COMT) gene: an Italian study on 87 cancer children and a systematic review
Source: BMC Cancer. 2019 Jan 31;19:113. doi: 10.1186/s12885-019-5310-4 (PMC6357360; doi:10.1186/s12885-019-5310-4)
Supplement: Supplementary file 4 — BMC Cancer.doc, Efficacy and safety parameters of 8 missing subjects in the STOP Pain Project. (DOCX 25 kb) [file 12885_2019_5310_MOESM4_ESM.docx]

**Table S4.** Efficacy and safety parameters of 8 missing subjects in the STOP Pain Project.

|  | **Overall** |
| --- | --- |
| Opioids |  |
| **Dose** (mg/kg), mean (±SD) |  |
| Dose_24h_ | 0.24 (±0.179) |
| Dose_tot_ | 1.95 (±1.406) |
| Dose_VAS=0_ | 0.20 (±0.141) |
| **Pain Intensity**, mean (±SD) |  |
| ∆ _VAS_ | 2.75 (±2.964) |
| Time _tot_ (hours) | 135.31 (±60.480) |
| **Side effects**, N (%)^a^ |  |
| Gastrointestinal^b^ | 3 (26.44) |
| CNS^c^ | 2 (11.49) |
| Total^d^ | 4 (36.78) |
