# Supplementary material for: Opioid response in paediatric cancer patients and the Val158Met polymorphism of the human catechol-O-methyltransferase (COMT) gene: an Italian study on 87 cancer children and a systematic review
Source: BMC Cancer. 2019 Jan 31;19:113. doi: 10.1186/s12885-019-5310-4 (PMC6357360; doi:10.1186/s12885-019-5310-4)
Supplement: Supplementary file 5 — BMC Cancer.doc, Characteristics of studies excluded after full-text reading. (DOCX 26 kb) [file 12885_2019_5310_MOESM5_ESM.docx]

**Table S5.** Characteristics of 15 studies excluded after full-text reading.

| **Study name, [Ref]** | **Study aim** | **Patients’ characteristics** | **Number of patients** | **Sex M/F** | **Mean Age (years±SD)** | **Reason for exclusion** |
| --- | --- | --- | --- | --- | --- | --- |
| Aouizerat, 2015 | To test for associations between SNPs in 5 opioid receptor genes and the occurrence of preoperative  breast pain | Women with breast cancer-related pre- and postoperative pain | 352 | 0/352 | NR | Conference abstract |
| Chou, 2006 | To determine morphine consumption with intravenous patient-controlled analgesia according to A118G polymorphism | Women who underwent abdominal total hysterectomy | 80 | 0/80 | 46±6 | Postoperative pain; no data on preoperative pain |
| Galvan, 2012 | To test for associations between one million SNPs and response to opioid therapy | European cancer patients with poor or good response to opioids | 1008 | NR | NR | Conference abstract for the included study by Galvan 2012 |
| Gonzaléz, 2015 | Observational, retrospective non-interventionist to determine efficacy and safety of treatment with opioids in relation to the polymorphism of OPRM1 | Patients with cancer and non-cancer related chronic pain | 190 | 128/62 | 57.7±14.2 | Conference abstract; no results for cancer patients subgroup |
| Kambur, 2013 | To examine the effect of COMT polymorphisms on experimental and postoperative pain phenotypes | Patients operated for breast cancer | 1000 | 0/1000 | 57.0±9.3 | Experimental and postoperative pain |
| Kleine-Brueggeney, 2010 | Review on clinical trials in pain patients genetic variants which have a possible impact on analgesics and adjuvant or anticancer drugs pharmacokinetics or pharmacodynamics | - | - | - | - | Review |
| Matsunaga, 2009 | To compare the serum concentrations of several pro-inflammatory cytokines and health-related quality of life between OPRM1 genotypes | Healthy volunteers | 123 | 65/58 | 25.4±1.24 | Study on healthy volunteers |
| Matsuoka, 2013 | To evaluate predictive biomarkers of the treatment outcome of morphine | Opioid-treatment-naïve cancer patients | NR | NR | NR | Conference abstract; study on pharmacokinetic parameters |
| Noble, 1998 | Comment on case-control study of the D2 dopamine receptor gene and smoking status in lung cancer patients | - | - | - | - | Editorial |
| Ochroch, 2013 | To investigate the genetic basis of elevations in acute postsurgical pain after  thoracotomy | Thoracotomy patients with diagnosed or suspected primary lung cancer | 90 | 48/42 | 68.9±9.4 | Postoperative pain, no data on preoperative period |
| Peiró Peiró, 2014 | Observational, prospective study focused on analgesic efficacy, opioid withdrawal syndrome prevention, adverse side effect, functional status and aberrant drug-related behaviour | Outpatients diagnosed with opioid iatrogenic dependence and severe pain intensity | 70 | NR | NR | Conference abstract; no cancer patients |
| Rudzianskiene, 2014 | To determine if the SNP of IL-6 and IL-10 cytokines could influence the analgesic response of radiotherapy in the treatment of painful bone destructions | Multiple myeloma patients | 30 | 11/19 | 67 (median) | Conference abstract; study on both palliative radiotherapy and opioid dose, no data on patients treated only with opioids |
| Skorpen, 2016 | To apply the “most likely candidate gene” approach to investigate the possibility that no or extraordinary poor response to high opioid doses is related to genetic defects | European cancer patients and Norwegian healthy volunteers | 2761 | NR | NR | Study on DNA sequencing |
| Wesmiller, 2016 | To describe the incidence and explore the risk  factors associated with postoperative nausea and vomiting | Women diagnosed with early stage breast cancer scheduled for surgery | 93 | 0/93 | 58.3±9.1 | Postoperative side effects; no data on preoperative period |
| Zhao, 2016 | To evaluate the impact of the 174G/C SNP of IL-6 on the prognosis and pain tolerance of non-small cell lung cancer (NSCLC) patients | NSCLC patients | 434 | 339/93  2 cases unknown | 64.0±10.25 | Retracted article |
